# Analysis of prenatal diagnosis before and after implementation of the two-child policy in northeastern China

**DOI:** 10.1097/MD.0000000000017200

**Published:** 2019-09-20

**Authors:** Ruixue Wang, Yang Yu, Qi Xi, Yuting Jiang, Haibo Zhu, Shibo Li, Ruizhi Liu, Hongguo Zhang

**Affiliations:** a Center for Reproductive Medicine and Center for Prenatal Diagnosis, First Hospital, Jilin University, Changchun, China; b Department of Pediatrics, Genetics Laboratory at University of Oklahoma Health Sciences Center, Oklahoma City, OK, USA.

**Keywords:** advanced maternal age, chromosomal abnormality, down syndrome, prenatal diagnosis, two-child policy

## Abstract

The universal two-child policy has now been fully implemented in China. This change requires adaptations to maternal care and childcare systems, but the features of prenatal diagnosis before and after implementation of the policy have not been reported.

We conducted a retrospective study of 6736 prenatal cytogenetic diagnoses performed on amniotic fluid cells over a 4-year period, including 2 years before and after implementation of the second child policy. Amniotic fluid cells collected through amniocentesis were cultured, harvested, and stained for chromosome analysis using standard laboratory protocols.

The study included 3222 pregnant women referred before implementation of the policy, which we used as a control group, and 3514 pregnant women referred after policy implementation as an investigational study group. There were significantly fewer pregnant women aged <25 years in the investigational group than in the control group (*P* < .001). There were no significant between-group differences for other pregnant women aged >31 years and 27–28 years old (*P* > .05). A total of 358 cases with chromosomal abnormalities were diagnosed, including 129 (4%, 129/3222) in the control group which was significantly lower than the 229 (6.5%, 229/3514) in the study group (*P* < .001). In particular, significantly more trisomy 21 cases were observed in the study group than in the control group (120 vs 59). More pregnant women underwent non-invasive prenatal testing (NIPT) in the study group (46%) than in the control group (20%). In the study group, the average age of pregnant women who underwent NIPT was significantly higher than that of women who did not receive NIPT (*P* < .05). However, there were no significant between-group differences for the control group (*P* > .05).

The number of cases with chromosomal abnormalities increased in northeastern China in the 2 years after implementation of the two-child policy. The number of pregnant women of advanced maternal age did not increase significantly, perhaps because of the widespread application of NIPT. However, the number of fetuses with Down syndrome increased significantly, suggesting that prenatal screening and diagnosis should be strengthened.

## 1 Introduction

The universal two-child policy has been fully implemented in China since January 2016.^[1]^ Some demographers predicted that this would greatly increase the birth rate in China, increase the number of pregnant women with advanced maternal age (AMA), and increase pressure on prenatal diagnosis. However, the number of births in 2016 and 2017 was 17.86 million and 17.23 million, respectively, reflecting a slight decrease in both the number of births and the birth rate in 2017. However, the new challenges facing prenatal diagnosis and pregnant women with AMA are worthy of attention.

An increase in the number of pregnant women with AMA is often accompanied by increased fetal chromosomal abnormalities.^[2]^ In Western Australia, a greater proportion of Down syndrome pregnancies from 1980 to 2013 were in women of AMA.^[3]^ Moreover, Kim et al^[4]^ reported that the risks of trisomy 21 and trisomy 18 are correlated with maternal age, and this remains a common risk factor for fetal chromosomal abnormalities.

The universal two-child policy requires adaptations to childcare systems and maternal care,^[5]^ including prenatal screening and diagnosis. However, the features of prenatal diagnosis before and after implementation of the two-child policy have not been reported. In the present study, therefore, we explored this in northeastern China.

## 2 Subjects and methods

### 2.1 Subjects and study design

This retrospective study was based on 6736 cases of prenatal cytogenetic diagnosis performed on amniotic fluid cells over a 4-year period (January 2014–December 2017) in the cytogenetic laboratories of the Center for Reproductive Medicine and Center for Prenatal Diagnosis, First Hospital of Jilin University. We compared patient age differences and chromosomal anomaly incidences detected prenatally before and after implementation of the two-child policy in northeastern China, which had an official start date of January 2016. All cases were divided into two groups. The control group (3222 cases) enrolled women from January 2014 to December 2015 (before implementation of the two-child policy), and the study group (3514 cases) enrolled women from January 2016 to December 2017 (after policy implementation). Genetic counseling was provided to each patient prior to prenatal diagnosis, including information about the benefits and disadvantages of amniocentesis and the testing process, and possible results and limitations of karyotyping. The study protocol was approved by the Ethics Committee of the First Hospital of Jilin University, and written informed consent was obtained from all participants.

### 2.2 Prenatal diagnosis of amniotic fluid cells

All patients underwent detailed ultrasonographic evaluation (including placental localization and fetus positioning) before amniocentesis. Amniocentesis was performed between 16 and 24 weeks’ gestation, and amniotic fluid was obtained by ultrasound-guided transabdominal aspiration.

Amniotic fluid samples were harvested into 15 mL centrifuge tubes and transferred directly to the laboratory for culture. Supernatants were discarded, and cell suspensions (1–1.5 mL) were collected after centrifugation at 252 g for 6 min. They were then used to inoculate 25 cm^2^ flasks with 5 mL culture medium (GIBCO Amnio MAX-II complete medium, Invitrogen Corporation, Carlsbad, CA, USA). Cell culture and G-banding for chromosomal sample preparation were performed using previously described methods.^[6]^ In all cases, at least 20 metaphase plates were counted and six karyotypes were analyzed for each patient. The karyotype nomenclature is described in accordance with the International System for Human Cytogenetic Nomenclature 2009.

### 2.3 Statistical analysis

Data were analyzed using SPSS software v18.0 (SPSS Inc., Chicago, IL, USA). Data were presented as means ± SD or n (%), and compared using independent sample tests or the χ^2^ test. All statistical tests were two-sided, and a *P* value of <.05 was considered to indicate statistical significance.

## 3 Results

The mean age of women in the control group (30.47 ± 5.58 years) was significantly lower than in the study group (30.88 ± 5.29 years; *P* < .05). Significantly fewer pregnant women in the study group were <25 years old and 25 to 26 years old compared with the control group (*P* < .001), while significantly more were aged 29 to 30 years old in the study group than in the control group (*P* < .01). There were no significant between-group differences for other pregnant women aged >31 years and 27 to 28 years old (*P* > .05) (Table 1).

**Table 1:**
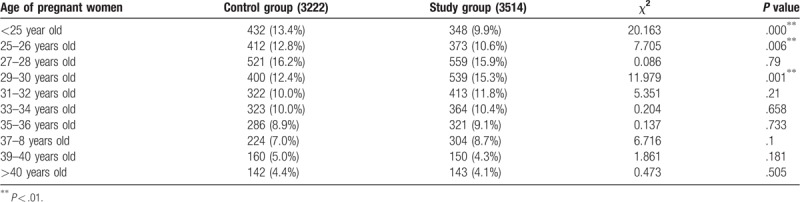
Changes in the age distribution of pregnant women.

The cases diagnosed with chromosomal abnormalities are summarized in Table 2. A total of 358 cases were diagnosed with chromosomal abnormalities, including 129 (36%, 129/358) in the control group and 229 (64%, 229/358) in the study group. This difference was significant (*P* < .001). Chromosomal abnormalities were significant differences between the control and study groups for pregnant women aged 25 to 26 years, 29 to 30 years old (*P* < .05) and 37 to 38 years old (*P* < .01). There were no significant between-group differences with respect to chromosomal abnormalities for other pregnant women aged <25 years, 27 to 28 years old, 31 to 36 years old, >39 years old (*P* > .05).

**Table 2:**
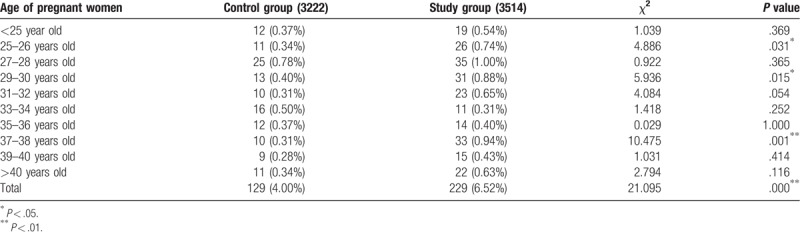
Changes in the number of cases of chromosomal abnormalities.

**Figure 1:**
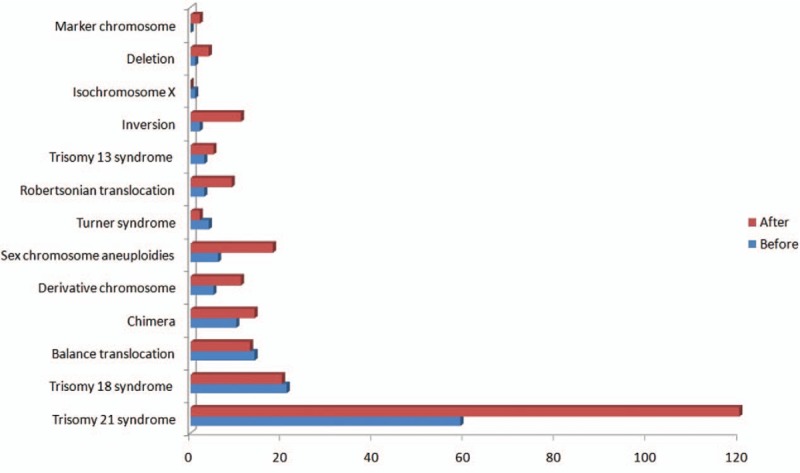
The type and number of cases with chromosomal abnormalities after and before implementation of the second-child policy.

The type and number of cases with chromosomal abnormalities are shown in Figure 1. Trisomy 21 syndrome was observed in 120 patients in the study group, which is significantly higher than seen in the control group (59 cases). The number of chromosomal inversions, Robertsonian translocations, sex chromosome aneuploidies, and derivative chromosomes were also significantly higher in the study group than in the control group (χ^2^ = 14.178, *P* < .001).

Considering that we found no increase in the number of older pregnant women, but the number of cases of Down syndrome significantly increased after implementation of the two-child policy, we further analyzed the extent of NIPT testing in pregnant women found to be carrying fetuses with Down syndrome. In this subgroup, more women underwent NIPT after policy implementation (46%) than before (20%). Moreover, after policy implementation, the average age of pregnant women who underwent NIPT was significantly higher than those who did not (*P* < .05) (Table 3). There were no significant between-group differences before policy implementation (*P* > .05) (Table 3).

**Table 3:**
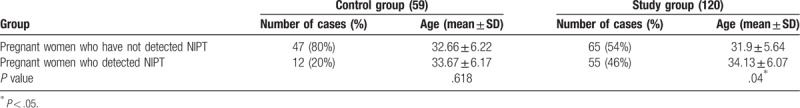
Pregnant women who underwent NIPT in all cases diagnosed with Down syndrome.

## 4 Discussion

In China, implementation of the two-child policy in 2016 resulted in important implications for maternity and child care. The culture of delivery decision-making has changed, leading to a decrease in cesarean delivery rates,^[7]^ with women intending to have a second child being more likely to reject cesarean section as their delivery method.^[8]^

In the present study, we compared prenatal diagnosis data from a 2-year period after the policy change with data from before the policy change. We revealed a significantly higher average age of pregnant women after the policy change than before. Moreover, a significantly lower number of pregnant women were aged <25 years and 25 to 26 years old after the policy change than before, but there was no significant increase in those aged >31 years and 27 to 28 years old. Zhang et al^[1]^ recently reported that 90 million couples met the conditions of the two-child policy, and that 60% of these couples were aged over 35 years. Although this may suggest that there is a large number of older pregnant women after implementation of the two-child policy, our data in fact show that the numbers have not increased.

Cytogenetic analysis remains a powerful and cheap technology, and continues to have wide applications in the field of genetics, including prenatal diagnosis.^[9]^ In laboratory testing, karyotyping has lower experimental failure rates than quantitative fluorescent PCR and array comparative genomic hybridization for pregnant women <24 gestational weeks.^[10]^ Karyotyping is also recommended by the American College of Obstetricians and Gynecologists (ACOG) and the Society for Maternal–Fetal Medicine (SMFM) for pregnant women who wish to pursue prenatal diagnosis in the setting of a normal fetal ultrasound.^[11]^

In this study, we reported changes in the type and quantity of chromosomal abnormalities before and after implementation of the two-child policy. Significantly more cases were diagnosed with chromosomal abnormalities after than before the policy change, and most of these cases were aged 37 to 38 years old. Women of AMA have an increased chance of developing a fetal abnormality.^[12]^ In female meiosis, incorrect chromosome segregation or unstable sister kinetochore attachments, which occur with increasing age, can result in the production of eggs prone to aneuploidy.^[13,14]^ Hence, AMA women in China are advised to undergo prenatal diagnosis, including fetal chromosomal analysis.^[12]^

In the present study, a total of 120 cases with Down syndrome were diagnosed in the 2-year period after implementation of the two-child policy, compared with only 59 in the 2 years before the policy change. Additionally, chromosomal inversions, Robertsonian translocations, sex chromosome aneuploidies, and derivative chromosomes increased after implementation of the two-child policy. To meet these new challenges, genetic counseling for prenatal diagnosis should be strengthened.

Although we found no increase in the number of older pregnant women after policy implementation in the present study, the number of fetuses with Down syndrome significantly increased. More pregnant women also underwent NIPT after the policy change than before, and testing was performed in women significantly older than those who did not undergo NIPT post-policy. These results showed that the application of NIPT testing may play an important role in not increasing the number of pregnant women with AMA. Neufeld-Kaiser et al^[15]^ reported 50% of frequent indications for NIPT were women of AMA. More pregnant women with AMA chose NIPT detection, and some low-risk individuals are excluded. This could result in a reduction in the number of pregnant women with AMA who underwent prenatal diagnosis. These also indicate that NIPT can change the age composition of pregnant women with prenatal diagnosis. Since the two-child policy was implemented, more pregnant women have applied to undergo NIPT than 2-year period before the policy change.

In general, pregnant Chinese women strongly request a full chromosomal assessment, which influences pregnancy outcome.^[16]^ In this study, pregnancy terminations were requested by women carrying fetuses with Down syndrome, trisomy 18 syndrome, trisomy 13 syndrome, Turner syndrome, deletions, and chimeras. When fetal marker chromosomes or derivative chromosomes exist, samples are further diagnosed by molecular genetics, allowing the couple to make an informed choice whether to continue the pregnancy. Similarly, in cases of fetal balance translocations, Robertsonian translocations, inversions, sex chromosome aneuploidies, or isochromosomes, couples are informed of the clinical characteristics, treatments or cures, and future fertility status.

A limitation of this study is the analysis of single-center data. To our knowledge, this is the first report on the impact after the implementation of the two-child policy in China on prenatal diagnosis. Further multicenter studies will provide beneficial value for the change of prenatal diagnosis strategy.

## 5 Conclusions

In summary, we observed an increase in the number of pregnant women carrying fetuses with chromosomal abnormalities in northeastern China in the 2-year period after implementation of the two-child policy than before. In particular, the number of fetuses with Down syndrome increased significantly. However, the number of pregnant women of AMA did not increase significantly, which may be related to the widespread application of NIPT. Nevertheless, we propose that prenatal screening and diagnosis should be strengthened.

## Author contributions

**Data curation:** Yang Yu.

**Funding acquisition:** Ruizhi Liu.

**Investigation:** Ruixue Wang.

**Methodology:** Qi Xi, Yuting Jiang.

**Software:** Haibo Zhu.

**Writing – original draft:** Ruixue Wang, Hongguo Zhang.

**Writing – review & editing:** Shibo Li, Hongguo Zhang.

Hongguo Zhang orcid: 0000-0001-8953-863X.
